# Activation of JNK Triggers Release of Brd4 from Mitotic Chromosomes and Mediates Protection from Drug-Induced Mitotic Stress

**DOI:** 10.1371/journal.pone.0034719

**Published:** 2012-05-02

**Authors:** Akira Nishiyama, Anup Dey, Tomohiko Tamura, Minoru Ko, Keiko Ozato

**Affiliations:** 1 Program in Genomics of Differentiation, National Institute of Child Health and Human Development, National Institutes of Health, Bethesda, Maryland, United States of America; 2 Department of Immunology, Yokohama City University Graduate School of Medicine, Kanazawa-ku, Yokohama, Kanagawa, Japan; 3 Section on Developmental Genomics and Aging, Laboratory of Genetics, National Institute of Aging, National Institutes of Health, Baltimore, Maryland, United States of America; Wellcome Trust Centre for Stem Cell Research, United Kingdom

## Abstract

Some anti-cancer drugs, including those that alter microtubule dynamics target mitotic cells and induce apoptosis in some cell types. However, such drugs elicit protective responses in other cell types allowing cells to escape from drug-induced mitotic inhibition. Cells with a faulty protective mechanism undergo defective mitosis, leading to genome instability. Brd4 is a double bromodomain protein that remains on chromosomes during mitosis. However, Brd4 is released from mitotic chromosomes when cells are exposed to anti-mitotic drugs including nocodazole. Neither the mechanisms, nor the biological significance of drug-induced Brd4 release has been fully understood. We found that deletion of the internal C-terminal region abolished nocodazole induced Brd4 release from mouse P19 cells. Furthermore, cells expressing truncated Brd4, unable to dissociate from chromosomes were blocked from mitotic progression and failed to complete cell division. We also found that pharmacological and peptide inhibitors of the c-jun-N-terminal kinases (JNK) pathway, but not inhibitors of other MAP kinases, prevented release of Brd4 from chromosomes. The JNK inhibitor that blocked Brd4 release also blocked mitotic progression. Further supporting the role of JNK in Brd4 release, JNK2–/– embryonic fibroblasts were defective in Brd4 release and sustained greater inhibition of cell growth after nocodazole treatment. In sum, activation of JNK pathway triggers release of Brd4 from chromosomes upon nocodazole treatment, which mediates a protective response designed to minimize drug-induced mitotic stress.

## Introduction

Anti-mitotic drugs that interfere with microtubule dynamics are used in cancer chemotherapy [1], [2]. These drugs, such as nocodazole, colcemid and taxol arrest cells at prometaphase, and induce rapid apoptosis in some cancer cells. However, these drugs also prompt activation of a protective mechanism in other cells, allowing cells to survive and go through mitosis [3]. A reversible anti-tubulin agent, nocodazole has been extensively investigated to study protective responses against mitotic stress, because nocodazole treated cells, upon drug removal, resume mitosis and produce viable daughter cells, although nocodazole treatment delays mitotic progression and increases aneuploidy and genome instability [1], [4].

Anti-mitotic drugs activate mitogen activated kinase (MAPK) pathways that regulate various stress responses, resulting in cell survival and/or death [5], [6], [7]. The c-jun NH2 terminal kinases (JNK), among other MAPKs are activated by anti-tubulin drugs in many cancer cells [6], [7], [8], [9], [10], [11]. Furthermore, there is evidence indicating that JNK is activated during the normal course of mitosis and plays a role in some stages of mitosis [10], [12], [13], [14], [15], [16]. Among three JNKs, JNK1 and JNK2 are ubiquitously expressed and thought to have distinct and overlapping roles in diverse settings. JNK3 is expressed in a brain specific manner [17], [18]. JNK appears to manifest complex, seemingly opposite biological activities in cancer and normal cells [19]. For example, JNK is associated with cell death as well as cell survival, since it elicits pro- and anti-apoptotic activities in a context dependent manner. Similarly, JNK is reported to have pro- and anti-oncogenic activities depending on model systems.

Brd4 is a member of the conserved BET family [20]. It binds to acetylated histone H3 and H4 through the two bromodomains present in the N-terminal region [21], [22]. As a salient feature of the BET family, Brd4 remains on chromosomes during mitosis in mammalian and zebrafish cells [21], [22], [23], [24], [25]. The retention of Brd4 and other BET proteins on mitotic chromosomes is unusual, given that most of general and specific transcription factors, even those with a bromodomain are released from chromatin during mitosis, leading to the general shut down of transcription [26], [27]. Besides the BET proteins, there are other proteins that remain bound on chromosomes during mitosis that act in epigenetic marking [28], [29]. Relevant to this, we found that Brd4, by staying on mitotic chromosomes, marks transcription start sites of genes programmed for early postmitoic transcription [30]. During interphase, Brd4 recruits a transcription elongation factor, P-TEFb and promotes expression of a large set of genes, thus regulating diverse biological activities [31], [32], [33], [34], [35], [36]. We previously showed that a variety of anti-tubulin drugs, including nocodazole, trigger complete release of Brd4 from mitotic chromosomes [37]. In that paper, we also reported evidence that Brd4 release is linked to cells’ recovery from drug-induced mitotic inhibition.

The aim of this study was to further investigate the potential link between Brd4 release and mitotic stress responses. To this end we addressed signaling pathways involved in Brd4 release and the functional significance of Brd4 release. Here we show by testing MAPK inhibitors, that activation of the JNK pathway is a major mechanism of nocodazole induced Brd4 release. Deletion analysis found that the C-terminal region of Brd4, unrelated to the bromodomains mediated its release. In line with the role for JNK, cells treated with a JNK inhibitor sustained greater impairment in mitotic progression after nocodazole treatment than without inhibitor. Matching with this result, cells expressing a Brd4 C-terminal deletion were defective in cell division after drug treatment. In addition, JNK2–/– embryonic fibroblasts were defective in drug-induced Brd4 release and endured greater growth inhibition than wild type cells. Together, our study supports the view that Brd4 release is triggered upon JNK activation, which leads to a protective response against drug-induced mitotic inhibition.

## Results

### Anti-tubulin and Other Anti-mitotic Drugs Trigger Release of Brd4 from Chromosomes

**Figure 1:**
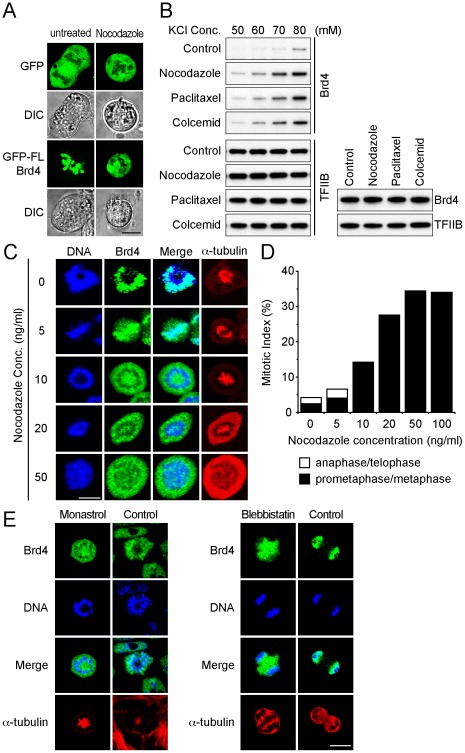
Anti-mitotic drugs induce release of Brd4 from mitotic chromosomes. A. P19 cells transfected with vectors for GFP alone or full length GFP-Brd4 (GFP-FL-Brd4) were treated with nocodazole (50 ng/ml) for 4 h, and GFP signals were viewed by confocal and differential interference contrast (DIC) microscopy. B. Differential salt extraction. Mitotic cells treated with nocodazole, paclitaxel or colcemid (100 ng/ml each) for 4 h were extracted with indicated concentrations of KCl. Twenty µg of extracts were immunoblotted with antibody for Brd4 or TFIIB. In the right panel, total Brd4 and TFIIB extracted at 400 mM KCl were detected as above. C. Cells were treated with indicated concentrations of nocodazole for 4 h, and mitotic cells were immunostained for endogenous Brd4 and α-tubulin. DNA was stained by Hoechst33324. D. Mitotic indices of nocodazole treated cells. Cells in prometaphase or anaphase/telophase were microscopically inspected. Approximately 200 cells were examined for each data point. E. Cells were treated with 100 µM of monastrol or 50 µM of blebbistatin for 4 h and localization of Brd4 was examined by immunostaining as in Figure 1C.

Persistent retention of Brd4 on mitotic chromosomes is a major feature of Brd4 in normal untreated cells. However, Brd4 is released from chromosomes upon treatment with anti-tubulin drugs [37]. Figure 1A shows live cell images of P19 cells expressing Brd4 fused to the green fluorescent protein (GFP-Brd4) with or without treatment with nocodazole. In untreated cells, the entire GFP-Brd4 localized to mitotic chromosomes (Figure S1A). In contrast, in nocodazole treated cells, Brd4 was entirely released from chromosomes into the outer space. In cells expressing free GFP, tested as a control, fluorescent signals were outside of chromosomes, as expected. Likewise, GFP-Brd4 was released from mitotic chromosomes when cells were exposed to other anti-tubulin agents, paclitaxel and colcemid (Figure S1B). Differential salt extraction experiments in Figure 1B showed that upon treatment with anti-tubulin agents Brd4 was eluted at salt concentrations lower than those observed in untreated cells. As shown in Figure 1B, the total amounts of Brd4 were unaltered by anti-tubulin drugs. These data provide microscopic and biochemical evidence that Brd4 is released upon treatment with anti-tubulin agents.

Since these agents inhibit mitotic spindle formation, we asked whether Brd4 is released as a result of disruption of spindle formation. It has been shown that these drugs at low concentrations do not break spindle mass formation, while arresting cells at prometaphase [2]. In Figure 1C, we tested the effect of nocodazole at 5 and 10 ng/ml, the doses lower than those required for disruption of spindle formation. At 5 ng/ml of nocodazole, Brd4 was partially released from mitotic chromosomes, while it was completely released at 10 ng/ml as verified by the separate localization of Brd4 and DNA (see the merge image). However, the architecture of mitotic spindles was well preserved at these concentrations. As expected, at higher nocodazole concentrations (20 and 50 ng/ml), spindle structures were altered or no longer recognizable. Data in Figure 1D show that mitotic arrest occurred both at 10 and 20 ng/ml of nocodazole treatment, albeit less efficiently than at 50 ng/ml. Thus, Brd4 release appeared not directly linked to spindle assembly disruption, suggesting the existence of other mechanisms controlling Brd4 release. To address whether Brd4 is released by anti-mitotic drugs that do not affect microtubule dynamics, we tested monasterol and Blebbistatin, small molecule inhibitors that impede mitotic processes by different mechanisms [38], [39]. Monasterol arrests cells at prometaphase by inhibiting kinesin, while blebbistatin blocks cytokinesis, a post anaphase event producing two daughter cells. Data in Figure 1E show that both agents also released Brd4 completely from chromosomes. Thus, release of Brd4 is a physiological response to a broad range of anti-mitotic drugs.

### Brd4 Release is Mediated by the Internal C-terminal Region

**Figure 2:**
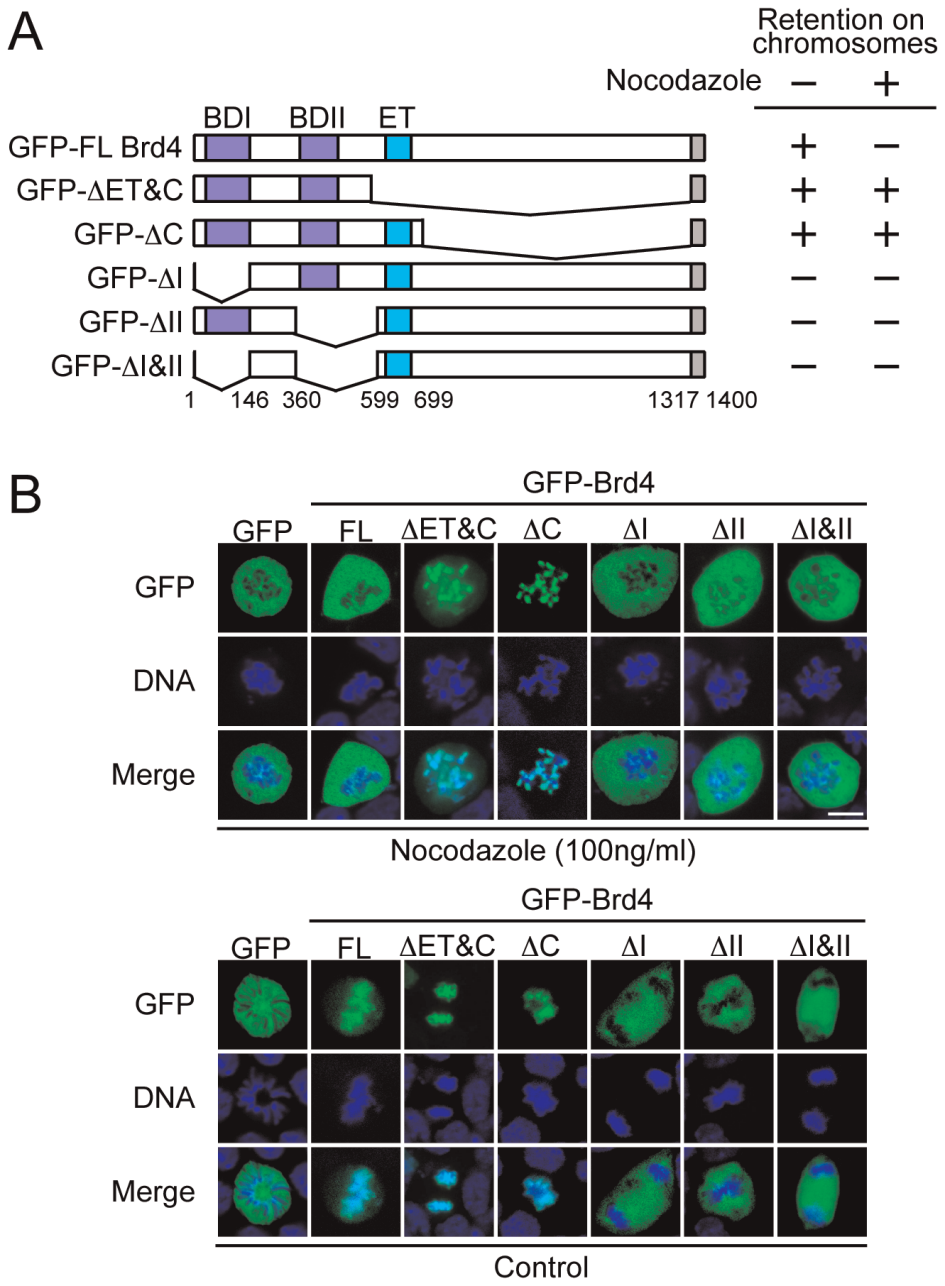
Domain analysis for nocodazole induced Brd4 release. A. Left: Diagram of Brd4 deletions. BDI and BDII represent bromodomains, ET stands for the ET domain. ΔET and ΔC are devoid of the internal C-terminal region, but contain the extreme C-terminal 83 amino acids. Right: A summary of nocodazole induced Brd4 release. + indicates retention of Brd4 on mitotic chromosomes, while– indicates Brd4 release after nocodazole treatment. B. Cells expressing free GFP, GFP-full length (FL) Brd4 or indicated deletions were treated with nocodazole (top panel) or vehicle alone (bottom panel) for 4 h and localization of GFP and chromosomes (DNA) was examined by microscopy. Approximately 200 mitotic cells were inspected for each deletion construct. After nocodazole treatment, more than 90% cells expressing ΔET&C and ΔC retained GFP signals on chromosomes, while less than 5% of full length Brd4 did so. Bromodomain deletions (ΔI, ΔII, and ΔI&II) and Free GFP did not localize to chromosomes, irrespective of drug treatment.

To assess domains within Brd4 that are required for nocodazole-induced Brd4 release, Brd4 deletions fused to GFP were expressed in P19 cells and tested for their localization after nocodazole treatment (see Figure 2A for a diagram of Brd4 deletion). Figure 2B illustrates representative images of the localization of each Brd4 deletion with (top) or without nocodazole treatment (bottom). Full length GFP-Brd4, while localizing to mitotic chromosomes in untreated cells, was released from chromosomes after treatment. Free GFP localized outside of chromosomes irrespective of drug treatment. In contrast, GFP-ΔET&C and GFP-ΔC were not released from chromosomes by the same treatment. These constructs lack the bulk of the internal C-terminal region, but retained the extreme C-terminal fragment from aa.1317 to aa.1400 (gray box in Figure 2A). The bromodomain deletions, ΔI, ΔII and ΔI & II did not localize to mitotic chromosomes and remained outside of the chromosomes with and without nocodazole treatment. The results with bromodomain deletions were expected, since binding of Brd4 to chromosomes depends on the bromodomains [21]. To quantify microscopic data, we counted approximately 200 cells for each construct, and confirmed that the images in Figure 2B represent more than 90% of cells (see summary in Figure 2A, right). These data show that the C-terminal region between aa. 700 to aa.1316 is critical for nocodazole induced Brd4 release. This region is relatively divergent among orthologues in different species, but contains a number of small motifs that are well preserved [22], [25]. In keeping with these results, Brd4 with an additional deletion lacking the extreme C-terminal fragment also failed to dissociate from chromosomes (data not shown). The requirement of the C-terminal region, not the bromodomains, indicates that nocodazole induced Brd4 release was not due to a change in Brd4’s acetyl histone binding activity.

### Brd4 Release Helps to Relieve Drug-induced Mitotic Inhibition

**Figure 3:**
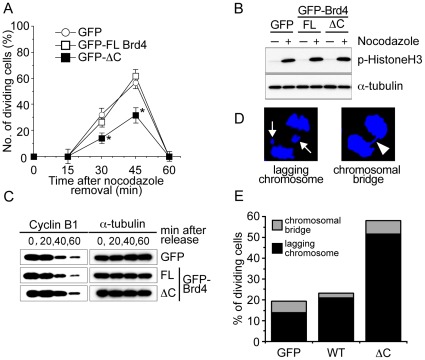
The Brd4 internal deletion (ΔC) inhibits mitotic progression after nocodazole treatment. A. Cells expressing free GFP, GFP-full length (FL) Brd4, or GFP-ΔC were treated with nocodazole for 4 h, washed and then allowed to proceed to anaphase and telophase in media without nocodazole for indicated times (min). The number of dividing cells with GFP signals were counted by microscopic inspection, and expressed as the percentage of total GFP positive cells. Similar experiments were performed for four times where mitotic cells with GFP-ΔC were consistently lower than those with GFP alone or GFP-FL Brd4. Extracts from cells expressing free GFP, GFP-FLBrd4, or GFPΔC treated with nocodazole were immunoblotted for H3 phosphorylated at S10. α-tubulin was tested as a loading control. *denotes p<0.005. B. Extracts from cells expressing above constructs were treated with nocodazole, and allowed to proceed through mitosis without nocodazole for indicated times (min), and immunoblotted for cyclin B1 and α-tubulin. C. Cells expressing above constructs were treated with nocodazole, washed and allowed to reach anaphase/telophase. Cells expressing GFP-ΔC were stained with Hoechst33324 to view chromosomal segregation. The images represent a typical lagging chromosome and chromosomal bridge, respectively. D. Mitotic cells expressing free GFP, GFP-FL-Brd4 or GFP-ΔC were inspected for chromosomal missegregation. Values represent the percentage of total dividing cells (approximately 200 cells per group).

To address the biological meaning of Brd4 release, we tested whether cells expressing GFP-ΔC were capable of going through mitosis after nocodazole treatment. In Figure 3A, cells expressing GFP-full length Brd4, free GFP or GFP-ΔC were first treated with nocodazole for 4 h, then nocodazole was removed by extensive wash. Cells were then allowed to proceed through mitosis in the following 60 min in fresh, drug free media. In Figure 3A, the number of mitotic cells (cells in anaphase and telophase) that carried GFP signals was counted at 15 min intervals. Cells expressing full length GFP-Brd4 and free GFP began entering anaphase/telophase at 30 min. The number of dividing cells peaked at 45 min where more than 50% of cells were in anaphase/telophase. Figure S2A is a representative image showing reloading of full length GFP-Brd4 on mitotic chromosomes after nocodazole removal. By 60 min, mitosis was completed and most cells were in G1 phase. In contrast, fewer GFP-ΔC expressing cells progressed to mitosis: only about 30% of cells were in anaphase/telophase at 45 min. By 60 min, virtually no mitotic cells were found in GFP-ΔC cells. These data suggest that Brd4 release is important for successful progression of mitosis after nocodazole treatment. To further assess a step affected by GFP-ΔC, we tested phosphorylation of histone H3 at Serine 10 and degradation of cyclin B1. These events denote entry into mitosis and progression through metaphase [40], [41], [42]. Immunoblot data in Figure 3B showed that H3 S10 phosphorylation occurred in cells expressing GFP-ΔC in a manner similar to those expressing GFP or full length GFP-Brd4. Similarly, cyclin B1 protein levels fell at 40 to 60 min, irrespective of the expression of full length Brd4 or GFP-ΔC (Figure 3C). These results indicate that expression of GFP-ΔC did not interfere with entry into mitosis, nor the initiation of exit from mitosis, but inhibited a subsequent step at anaphase/telophase.

Nocodazole treatment causes chromosomal missegregation, leading to genome instability in some cells [4]. Since anaphase/telophase is a stage when chromosomes begin to be segregated and partitioned into daughter cells, we examined whether GFP-ΔC expression affects chromosomal segregation. Microscopic images in Figure 3D and S2B illustrate lagging chromosomes and chromosomal bridges, representative defects noted for nocodazole treatment [4]. As shown in Figure 3E, the number of cells exhibiting defective chromosomal segregation was higher in cells expressing GFP-ΔC than those expressing full length GFP-Brd4 or free GFP. Nearly 60% of cells expressing GFP-ΔC were found to have chromosomal missegregation, the majority of them showing lagging chromosomes. About 20% of cells expressing free GFP or full length GFP-Brd4 also had abnormal chromosomal segregation, as expected [4].

Extensive mitotic detects observed with GFP-ΔC was somewhat surprising, given that these cells also expressed the endogenous, full length Brd4. The defect observed with GFP-ΔC may be attributed to a dominant negative activity of GFP-ΔC: we found that GFP-ΔC, but not full length GFP-Brd4, blocked release of full length Flag-tagged Brd4 from chromosomes (Figure S3). This dominant negative effect may be attributed to the interaction of full length Brd4 with ΔC that may occur through the bromodomains or by indirect mechanisms [43], [44], [45]. Thus, the marked defects observed with GFP-ΔC may partly be due to the concurrent inhibition of release of full length Brd4.

### Nocodazole-induced Brd4 Release Depends on Activation of the JNK Pathway

**Figure 4:**
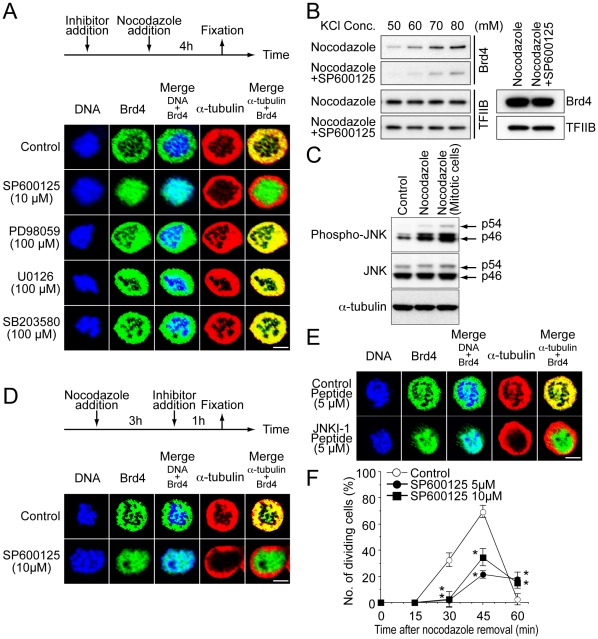
JNK inhibitors block nocodazole induced Brd4 release and prevent mitotic progression. A. SP600125 (10 µM), PD98059, U0126 or SB20458 (100 µM) was added to cells 30 min or 2 h prior to nocodazole addition, respectively. The inhibitors were present during the subsequent 4 h of nocodazole treatment (see top for the treatment scheme). Representative immunostaining data are shown below, depicting the localization of endogenous Brd4 and α-tubulin. Of about 200 SP600125 treated cells inspected, more than 90% of cells retained Brd4 on mitotic chromosomes after nocodazole treatment. B. Cells were treated with SP600125 and nocodazole as above and tested for differential salt extraction as in Figure 1B. C. Extracts from cells treated with or without nocodazole were blotted against total JNK or phosphorylated JNK. α-tubulin was tested as a loading control. The right lane represents samples from mitotic cells collected by mitotic shake-off. D. SP600125 was added to cells that had been treated with nocodazole for 3 h. Cells were incubated for an additional 1 h in the presence of the inhibitor and nocodazole before harvest, and immunostained as in Figure 4A. Of 200 mitotic cells counted, about 85% of cells treated with SP600125 and nocodazole retained Brd4 on chromosomes, while less than 10% of cells treated with nocodazole alone did. E. Cells were incubated with of control or JNKI-1 peptides (5 µM) for 30 min prior to 4 h nocodazole treatment. About 80% of JNKI-1 treated cells did not release Brd4 from chromosomes, while 90% of cells treated with the control peptide released Brd4. F. Cells treated with SP600125 and nocodazole using the scheme in Figure 4A were allowed to proceed through mitosis for indicated times (min), and stained with Hoechst33324 and cells in anaphase/telophase were counted as dividing cells. The values represent the average of three determinations +/– S.D. * indicates p<0.005.

Anti-mitotic drugs activate mitogen activated kinase (MAPK) pathways, including those for extracellular signal regulated kinases (ERK), p38, and JNK [5],[6],[7],[8]. To investigate whether a specific MAPK pathway is involved in nocodazole-induced Brd4 release, we tested pharmacological inhibitors of MAPKs. PD98059 and U0126 inhibit activity of MEK in the ERK pathways, and SB203580 inhibits p38 MAP kinase. SP600125 has been used as a specific inhibitor of JNK [46], [47], [48], [49]. These inhibitors were added prior to nocodazole addition and present during the next 4 h of nocodazole treatment. Localization of Brd4 was examined at the end of this treatment by immunostaining (Figure 4A). The inhibitors for MEK and p38 MAP kinase pathways had no effects on nocodazole-induced Brd4 release. In contrast, the JNK inhibitor, SP600125 completely blocked Brd4 release at concentrations ranging from 5 µM to 30 µM (Figure 4A, Figure S4A). The effect of the JNK inhibitor was particularly evident in the merge images where Brd4 colocalized with DNA, but not tubulin. On the other hand, in cells treated with other inhibitors and untreated cells, Brd4 showed an opposite pattern of colocalization, i,e., colocalizing with tubulin, but not with DNA. Of more than 200 mitotic cells inspected, approximately 85% of SP600125 treated cells showed Brd4 on chromosomes. Despite that the JNK inhibitor has a striking effect on Brd4 localization, it did not change nocodazole-induced spindle disruption (see α-tubulin staining), consistent with the earlier data in Figure 1C. In the absence of nocodazole, the inhibitor did not change Brd4’s localization to mitotic chromosomes, indicating that the inhibitor altered the movement of Brd4 only in nocodazole treated cells, but not untreated mitotic cells (Figure S4B). These data gave a first clue for the role of JNK pathways in Brd4 release. The inhibition of Brd4 release by SP600125 was further substantiated by differential salt extraction data, where the inhibitor reduced the amounts of Brd4 extracted at KCl concentrations ranging from 50 mM to 80 mM (Figure 4B left). Extraction of TFIIB, tested as a control, was not affected by SP600125. Similarly, the total levels of Brd4 or TFIIB were unaltered by SP600125 (Figure 4B, right). Since these data pointed to a role for JNK activation in Brd4 release, we next tested whether JNK was activated after nocodazole treatment in these cells. Immunoblot analysis with antibody against phosphorylated JNK showed a marked increase in phosphorylated JNK after nocodazole treatment, while total JNK levels were unchanged by the drug treatment (Figure 4C).

Because SP600125 was added before nocodazole treatment in above experiments, we next examined whether SP600125 inhibits Brd4 release when added after nocodazole treatment. In Figure 4D and S4C, cells were treated with nocodazole for 3 h and then treated with SP600125 for the remaining 1 h. The delayed addition of the inhibitor also caused inhibition in Brd4 release, indicating that the inhibitor exerts its effect rapidly, even after nocodazole treatment.

To further corroborate the role of JNK, another JNK inhibitor, JNKI-1 was tested [50]. This inhibitor is a cell penetrable peptide derived from the JNK interacting protein 1/Islet -brain1 (JIP-1/IB1) that blocks binding of substrates to the enzymes. As shown in Figure 4E and S4D, JNKI-1 also inhibited nocodazole-induced Brd4 release. Similar to SP600125, spindle disruption was not affected by the inhibitor. As expected, control peptide did not inhibit nocodazole-induced Brd4 release. Together, these data indicate that activation of the JNK pathway accounts for nocodazole-induced Brd4 release.

In light of the data in Figure 3A showing that inhibition of Brd4 release leads to inhibition of mitosis, we surmised that inhibition of JNK activity may also cause inhibition of mitotic progression. To test this possibility, cells were pretreated with 5 or 10 µM of SP600125 followed by 4 h of nocodazole treatment. Then nocodazole was removed from media allowing cells to proceed through mitosis. In Figure 4F, mitotic progression was quantified by counting anaphase and telophase cells at various time points. As observed in Figure 3A, nocodazole treated cells without inhibitor began dividing at 30 min. The number of dividing cells peaked at 45 min where more than 60% of cells were in cell division (open circle in Figure 4F). In contrast, the number of dividing cells was markedly reduced in cells treated with SP600125 at 5 µM and 10 µM: in the presence of the inhibitor, only 20 to 33% of cells were in cell division (Closed symbols in Figure 4F). Thus, the inability of releasing Brd4 from chromosome again correlated with the inhibition of cell division. Together, these data indicate that JNK activation triggers Brd4 release, which prompts a protective response against nocodazole induced mitotic inhibition.

### JNK2–/– Fibroblasts are Defective in Brd4 Release and Sustain Growth Inhibition After Nocodazole Treatment

**Figure 5:**
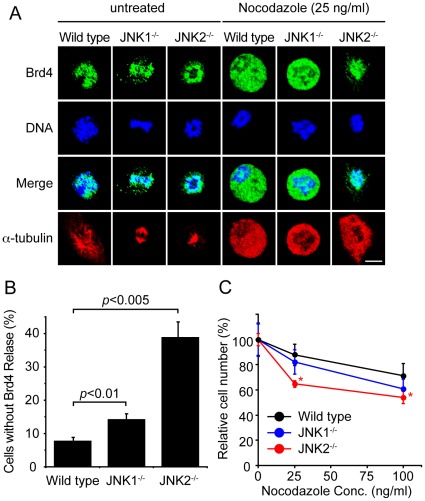
JNK2–/–MEFs are impaired in Brd4 release after nocodazole treatment. A. Wild type, JNK1–/– and JNK2–/– MEFs were treated with nocodazole at 25 ng/ml for 4 h. Endogenous Brd4, α-tubulin and DNA were localized by immunostaining as above. A greater number of JNK2–/– cells failed to release Brd4 after nocodazole treatment than wild type and JNK1–/– cells. B. Mitotic cells that failed to release Brd4 after nocodazole treatment in above experiments were quantified and expressed as the percentage of total mitotic cells. The values represent the average of three determinations +/– S.D. C. MEFs were treated with indicated concentrations of nocodazole for 6 h, washed and allowed to proceed for 36 h in culture, and total viable cell yields were determined. The values represent the average of three separate experiments +/– S.D. * indicates p<0.005.

To further investigate the role of JNK in Brd4 release, we next tested embryonic fibroblasts (MEFs) from JNK1–/– and JNK2–/– mice [51], [52]. In Figure 5A, JNK1–/–, JNK2–/– and wild type MEFs were treated with nocodazole and localization of endogenous Brd4 was examined by immunostaining. These experiments were performed using cells within four passages after primary culture. In untreated cells, Brd4 localized to mitotic chromosomes in all three cells. In wild type cells, Brd4 was completely released upon nocodazole addition. However, a large fraction of JNK2–/– cells retained Brd4 on chromosomes after nocodazole treatment. On the other hand, fewer JNK1–/– cells retained Brd4. Spindle formation was completely disrupted in all three cells, confirming nocodazole action in these cells. Data in Figure 5B show the number of mitotic cells that failed to release Brd4 after nocodazole treatment. More than 40% of JNK2–/– cells failed to release Brd4 from chromosomes upon drug treatment, while only ∼15% of JNK1–/– cells and ∼8% of wild type cells, respectively failed to release Brd4. These data indicate that JNK2 plays a somewhat dominant role over JNK1 in releasing Brd4, although both contribute to it.

To study whether the absence of JNK1 or JNK2 compromises recovery from drug-induced mitotic inhibition, nocodazole treated JNK1–/– and JNK2–/– cells were allowed to proceed for 36 h and viable cell yields were assessed at the end of culture (Figure 5C). In JNK2–/– cells, nocodazole treatment at 25 ng/ml caused approximately a 36% reduction in cell yields relative to untreated cells. On the other hand the same treatment led to a more modest reduction in JNK1–/– cells (12–17%). As expected, wild type cells showed the least reduction in viable cell yields. Similarly at a higher nocodazole concentration (100 ng/ml), viable cell yields were the lowest in JNK2–/– cells, the intermediate in JNK1–/– and the highest in wild type cells. These data support the idea that although both JNKs contribute, JNK2 plays a greater role in releasing Brd4 as well as restoring mitotic progression after nocodazole treatment than JNK1.

## Discussion

In this study we addressed the mechanism by which anti-mitotic drugs triggers release of Brd4 from mitotic chromosomes. Analysis of deletion constructs found that the internal region from aa. 670 to aa.1317 within the C-terminal domain is required for Brd4 release. This region is separate from the conserved bromodomains and the ET domain, and carries a histidine tract, several glutamine repeats and is rich in serine and proline [22], [25]. Since this region excludes the binding site for P-TEFb, important for transcription elongation, nocodazole induced Brd4 release is unrelated to Brd4’s interaction with P-TEFb [53]. In line with this conclusion, the interaction of Brd4 with P-TEFb is limited to interphase, in that the core component of P-TEFb, cyclin T and Cdk9 are released from chromatin during the normal course of mitosis [30]. We found that GFP-ΔC prevented the co-existing full length Brd4 to dissociate from chromosomes, suggesting that the truncated Brd4 acts as a dominant factor to reinforce its negative effect on full length Brd4. Although the underlying mechanism is not fully clear, a direct or indirect interaction between ΔC and full length Brd4 may explain the dominant negative effect [43], [44], [45].

Mitotic inhibition observed with ΔC may have a broader implication, since some cells express a truncated Brd4 similar to this truncation [54]. The inability of GFP-ΔC to dissociate from chromosomes correlated with abnormal chromosomal segregation and inhibition of mitotic progression. These data support the physiological significance of Brd4 release in controlling drug-induced mitotic stress. Pharmacological and peptide JNK inhibitors, when added prior to and during nocodazole treatment led to complete blockade of Brd4 release, which then led to defective mitotic progression, similar to that seen with ΔC. These results support the idea that JNK acts as a critical mediator of Brd4 release and helps to protect cells against drug-induced mitotic damage. However, this “protective” activity may create an adverse condition in some cells, namely increased drug resistance in cancer chemotherapy, a realistic possibility, given that anti-mitotic drugs such as taxol and vinblastine are often used for cancer treatment [1], [2].

It has been well documented that anti-tubulin drugs cause activation of JNK and other MAP kinase pathways [5], [6], [7]. Recent evidence indicates that JNK is activated during normal mitosis as well, and controls mitotic progression [12], [14], [15]. In some cells, JNK is reported to mediate histone H3 phosphorylation at serine 10 and activation of Cdk1 to downregulate cyclin B1 [12], [15]. Consistent with the role for JNK in mitosis, MKK7, an upstream kinase that activates JNK is shown to regulate G2/M phase of cell cycle, and affects cell proliferation and senescence [16]. However, since Brd4 is released only after drug treatment, not during normal course of mitosis, Brd4 release is not a part of JNK activation in normal mitosis, but it occurs as a result of drug-induced JNK activation. If JNK is activated in normal mitosis, why is Brd4 not released during normal mitosis? The seeming inconsistency may be readily explained by a quantitative threshold effect. Anti-mitotic drugs and other stresses appear to activate JNK at higher levels than in normal mitosis [9]. It is reasonable to consider that Brd4 release is triggered only when JNK activity reaches above a certain threshold. A similar, stress-dependent effect of JNK activity is reported for activation of apoptotic deal pathway [55] JNK is activated by many stress signals, which results in phosphorylation of a large set of substrates, resulting in the regulation of diverse biological activities [17], [18]. In light of the rapidity with which nocodazole and JNK inhibitors affect Brd4 release, it is possible that Brd4 is a canonical JNK substrate, and Brd4 is released from chromosomes due to the phosphorylation. Supporting this possibility, some serine residues in the Brd4 C-terminal region conform to the predicted phosphorylation sites for MAP kinases. However, it has been difficult for us to detect nocodazole induced Brd4 phosphorylation, partly because Brd4 is constitutively phosphorylated, and nocodazole induced changes, if they occur, are likely to be quantitative and subtle. In the absence of definitive results, it remains possible that Brd4 release is mediated by an indirect mechanism, rather than direct phosphorylation.

It is worth noting here that some of the changes previously attributed to JNK activation may not hold: a number of studies utilized SP600125 as a sole inhibitor to assess the function of JNK. However, this inhibitor is shown to have biological activities unrelated to JNK [56]. It is of note that activation of JNK produces seemingly opposite outcomes in some cases [19]: For example JNK activation is reported to promote apoptosis in some cases, while it is linked to cell survival in other cells [8], [57]. Furthermore, the literature indicates that JNK pathways regulate mitotic progression in a cell type and context dependent manner: while JNK is reported to control entry into mitosis, MacKorcle and Tan reported that JNK controls post-metaphase events, such as chromosomal segregation, without affecting earlier events such as cyclin B/Cdk1 activity [12], [13], [15]. The regulation of post-metaphase events was attributed to JNK2, not JNK1. This report is interesting, since defects we observed with ΔC and JNK inhibitors also concern anaphase/telophase events rather than earlier mitotic events. We also found that JNK2–/– MEFs manifest a greater deficiency in releasing Brd4 and they sustain greater cell growth inhibition than JNK1–/– cells. These results suggest that JNK2 plays a more dominant role in regulating Brd4 release and protecting against mitotic stress than JNK1. However, since JNK1–/– cells were also defective in mitotic progression, albeit to a lesser degree than JNK2–/– cells, it is likely that both JNK1 and JNK2 are at work in Brd4 release. This possibility is in line with the overlapping and distinct roles of the two JNKs reported before [58]. We noted that the defects found with either JNK1–/– and JNK2–/– cells were milder than those detected by ΔC or JNK inhibitors. This may be due to a compensatory mechanism activated in these knockout cells that can lessen the effect of gene disruption. Supporting this possibility, it has been reported that JNK2–/– cells express increased levels of JNK1 over wild type cells [59]. Further efforts to study the effect of JNK reexpression in the JNK–/– cells were unsuccessful, due to increased cell death (data not shown).

A significant question that arises from this study, which still awaits further investigation is how Brd4 release leads to protection against drug induced mitotic stress. A possible answer may lie in the Brd4’s function during mitosis [30]: we have shown that during mitosis the bulk of Brd4 binds to the transcription start sites of many, but not all RNA polymerase II dependent genes. These transcription start sites carry acetylated histone H3 and H4. Significantly, Brd4-marked genes are transcribed immediately after mitosis. It is suggested that orderly Brd4 release is required for the restoration of mitotic programs which needs to be established in response to exposure to anti-mitotic drugs, allowing cells to properly resume transcription in newly devided cells.

In conclusion, the chromatin binding protein Brd4 is released from chromosomes upon exposure to anti-mitotic drugs in a manner dependent on the activation of JNK pathway. JNK activation and Brd4 release may be a part of physiological responses designed to minimize drug-induced mitotic stress.

## Materials and Methods

### Ethics Statement

All animal experimentations were conducted in accordance with NIH and Public Health Service (PHS) policy. All protocols were approved by The Eunice Kennedy Shriver NICHD Animal Care and Use Committee (Protocol #08-010).

### Cell Culture

P19 embryonal carcinoma cells [22], obtained from American Tissue Culture Collection were maintained in alpha minimal essential medium with 10% fetal bovine serum supplemented with penicillin and streptomycin. JNK1 +/– and JNK2–/– mice (B6.129S1-Mapk8^fm1flv^/J and B6.129S2-Mapk9^tm1flv^/J) were obtained from Jackson Laboratories. Mouse embryonic fibroblasts (MEF) from JNK1–/– or JNK2–/– mice were prepared from embryos of day 14.5 p.c. and cultured in Dulbecco’s modified Eagle’s medium supplemented with 10% fetal bovine serum and used within four passages.

### Reagents and Plasmids

Nocodazole and other anti-mitotic drugs were purchased from Sigma-Aldrich. SP600125, SB 203580, PD 98059, U0126, and the JNK peptide inhibitor, JNKI-1 and control peptide were obtained from Calbiochem. Cells were treated with these inhibitors prior to and/or during nocodazole treatment as indicated in Figure Legends. Full length Flag-Brd4, full length GFP-Brd4 and internal deletions in pEGFP-C1 vector were described [60].

### Transfetion and Live Cell Microscopy

P19 cells (10^5^) were incubated in a 10 cm dish overnight and transfected with 10 µg of full length GFP-Brd4 or GFP-Brd4 deletions by Lipofectamine-Plus (Invitrogen). Twenty four h after transfection, cells were treated with nocodazole for 4–6 h at 100 ng/ml, mitotic cells were harvested by mitotic shake-off, stained with Hoechst 33342 and viewed on the Leica TCS-SP2 confocal microscope.

### Differential Salt Extraction

Mitotic cells collected after nocodazole treatment were suspended (5×10^4^ cells) in polyamine buffer (15 mM Tris-HCl, pH 7.5, 0.2 mM spermine, 0.5 mM spermidine, 2 mM EDTA, 20 mM KCl, and 0.1% digitonin) with protease inhibitor cocktail (Roche Diagnostic), homogenized, and centrifuged. Pellets were incubated with 60, 80, 100, 120 mM of KCl diluted in polyamine buffer for 30 min at 4°C with gentle rotation. Extracted proteins were resolved on 4–12% SDS PAGE (Invitrogen), transferred to Immobilon membrane (Millipore) and immunoblotted with anti-Brd4 antibody or anti-TFIIB antibody (Santa Cruz). Samples extracted with 400 mM KCl were used to detect total amounts of Brd4 and TFIIB.

### Immunostaining and Immunoblot Analysis

To localize endogenous Brd4, P19 and JNK–/– cells treated with anti-mitotic agents were cytospun, fixed, permeabilized and stained with antibodies for Brd4 and α-tubulin (Sigma-Aldrich) and counterstained with Hoechst33324 as described [37]. Whole cell extracts were prepared from 10^6^ cells using NP-40 buffer (50 mM Tris-HCl, pH 8.0, 150 mM NaCl, 1.0% NP-40, the protease inhibitor cocktail and phsophatase inhibitors (50 mM sodium fluoride, 1 mM sodium orthovanadate, and 0.1 mM ammonium molybdate)). Twenty µg of cleared extracts were subjected to immunobot analysis as above. Immunoblot detection of cyclinB1 was performed as described [37]. Phosphorylated histone H3 was detected by immunoblot analysis with samples extracted by RIPA buffer (20 mM Tris-HCl, pH 7.5, 150 mM NaCl, 1.0% NP-40, 1.0% Na deoxycholate, 0.1% SDS and protease inhibitor cocktail ) using antibody against serine 10 phopshorylated histone H3 (Millipore).

## Supporting Information

Figure S1 **A**: Localization of Brd4, DNA and alpha-tubulin on mitotic chromosomes of P19 cells undergoing mitosis. **B**: Distribution of Brd4, DNA and alpha-tubulin in P19 cells treated with nocodazole, paclitaxel and colcemid (each at 100 ng/ml) for 4 hours and untreated control during mitosis.
(TIF)

Figure S2 **A**: Reloading of Brd4 on mitotic chromosomes after nocodazole removal. Cells were treated with nocodazole for 4 hours at 100 ng/ml. Mitotic cells were then incubated in fresh media for indicated times. Cells were immunostained and counterstained for DNA. **B**: Association of Brd4 and delta-C mutant on mitotic chromosomes after nocodazole removal. P19 cells were transfected with GFP alone, GFP full length Brd4 or GFP delta-C and treated with nocodazole for 4 hours. At 45 min after nocodazole removal cell were fixed and stained for DNA. Arrowhead: chromosomal bridge.
(TIF)

Figure S3 P19 cells were cotransfected withFlag-tagged full length Brd4 along with either GFP alone, GFP-FL Brd4 or GFP-delta C and treated with nocodazole for 4 hours. Cells were fixed immunostained for Flag (red), GFP (green) and counterstained for DNA (blue).
(TIF)

Figure S4 **A**: JNK inhibitors block nocodazole induced Brd4 release. P19 cells were treated with various inhibitors 30 min or 2 hours prior to the addition of nocodazole. Treatment scheme is shown on the left. Cells were immunostained for localization of Brd4 and tubulin and counterstained for DNA. **B**: Inhibitors alone do not alter Brd4 localization on Mitotic chromosomes. P19 cells were treated with SP600125 or vehicle control prior to fixation and immunostaining. **C**: later treatment of inhibitors blocks nocodazole induced Brd4 release. SP600125 was added to cells 3 hours after the start of nocodazole treatment. Treatment scheme is shown on the left. **D**: JNKI-1 peptide blocks nocodazole induced Brd4 release from mitotic chromosomes. P19 cells were incubated with control or JNKI-1 peptides (5 µM) for 30 min prior to 4 hours nocodazole treatment. After the treatment, cells were immunostained for localization of Brd4, and alpha-tubulin and counterstained for DNA.
(TIF)
