# Supplementary figures and images for: Activation of JNK Triggers Release of Brd4 from Mitotic Chromosomes and Mediates Protection from Drug-Induced Mitotic Stress
Source: PLoS One. 2012 May 2;7(5):e34719. doi: 10.1371/journal.pone.0034719 (PMC3342290; doi:10.1371/journal.pone.0034719)

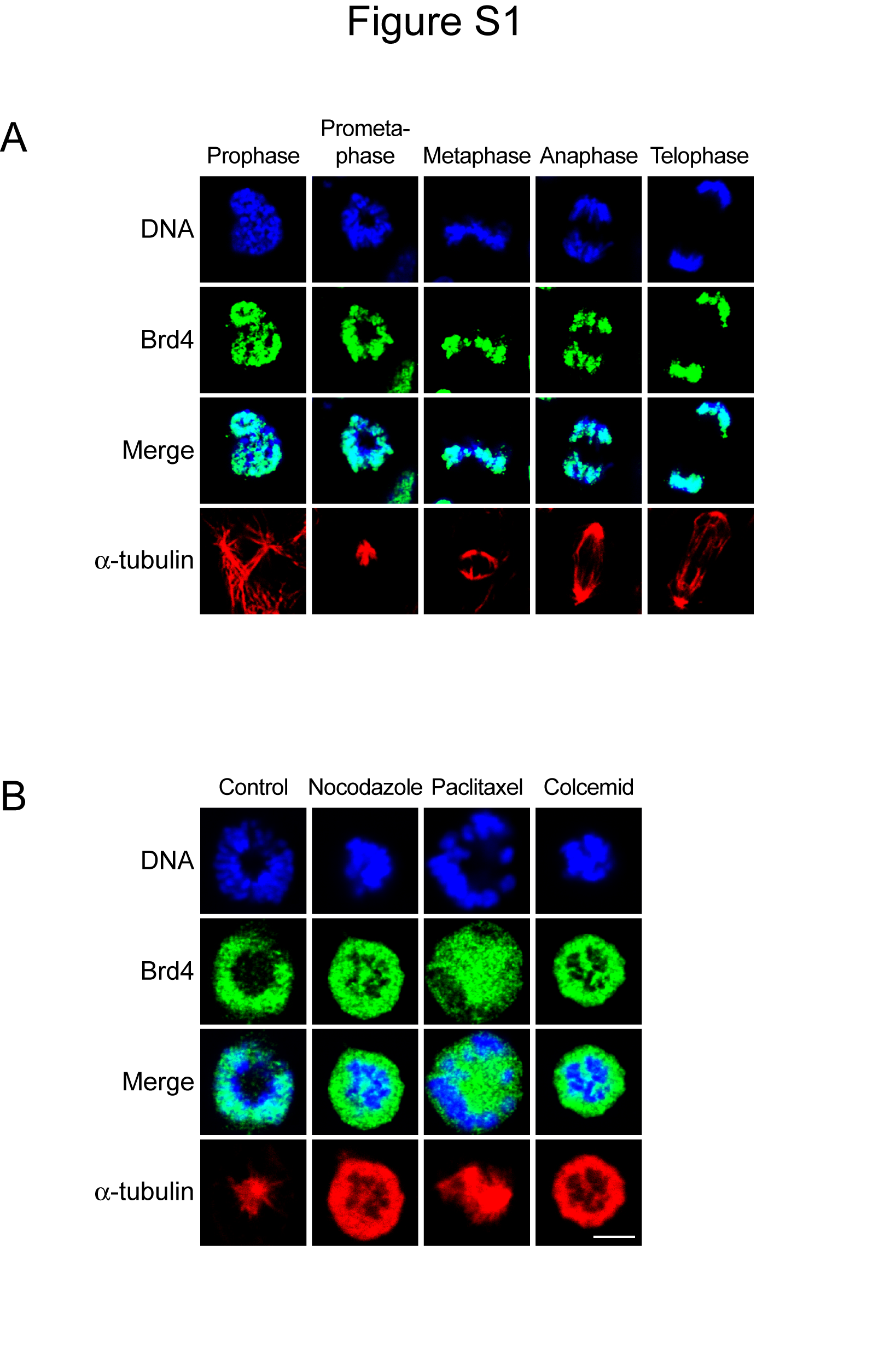

Supplement: Figure S1 — A: Localization of Brd4, DNA and alpha-tubulin on mitotic chromosomes of P19 cells undergoing mitosis. B: Distribution of Brd4, DNA and alpha-tubulin in P19 cells treated with nocodazole, paclitaxel and colcemid (each at 100 ng/ml) for 4 hours and untreated control during mitosis. (TIF) [file pone.0034719.s001.tif]

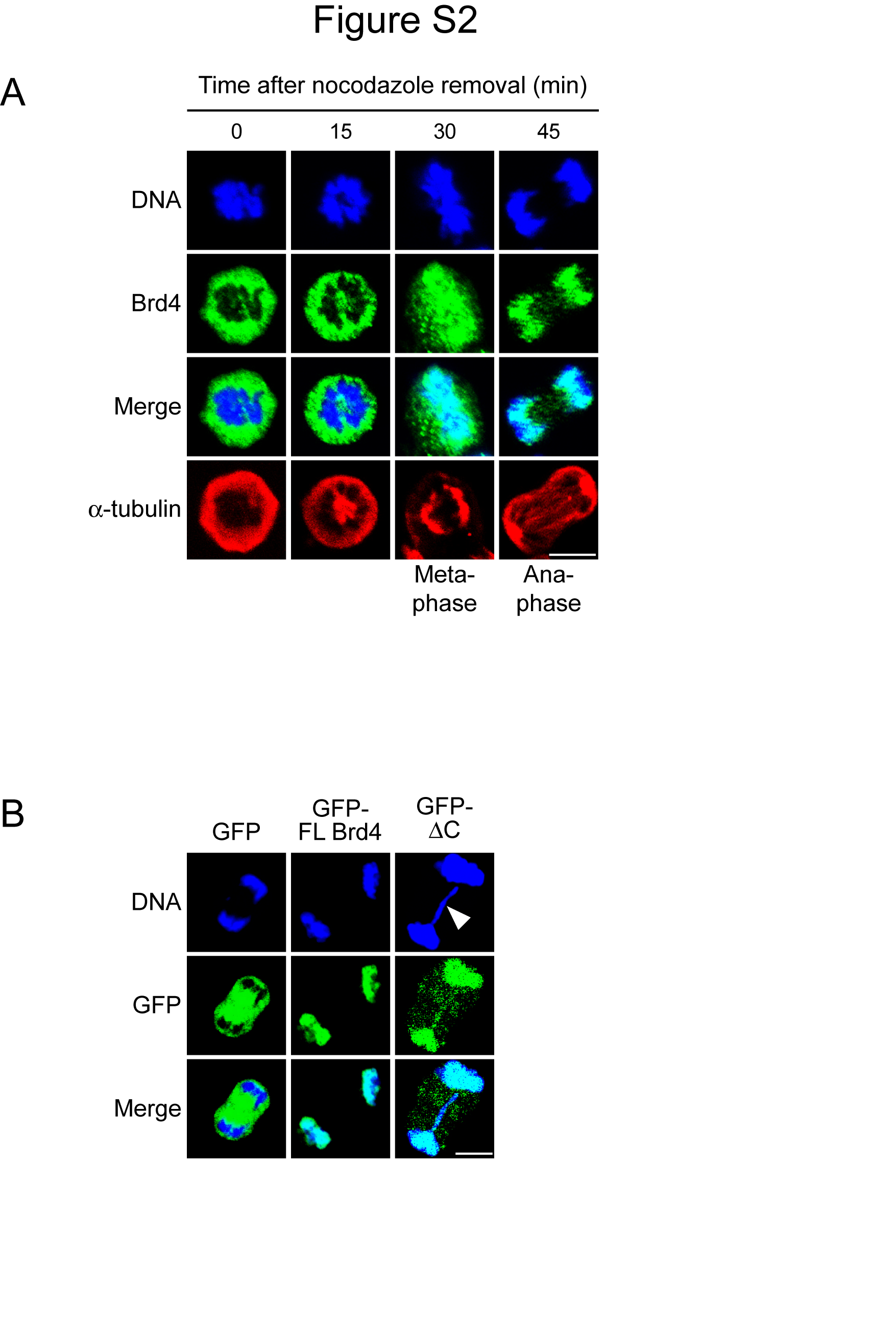

Supplement: Figure S2 — A: Reloading of Brd4 on mitotic chromosomes after nocodazole removal. Cells were treated with nocodazole for 4 hours at 100 ng/ml. Mitotic cells were then incubated in fresh media for indicated times. Cells were immunostained and counterstained for DNA. B: Association of Brd4 and delta-C mutant on mitotic chromosomes after nocodazole removal. P19 cells were transfected with GFP alone, GFP full length Brd4 or GFP delta-C and treated with nocodazole for 4 hours. At 45 min after nocodazole removal cell were fixed and stained for DNA. Arrowhead: chromosomal bridge. (TIF) [file pone.0034719.s002.tif]

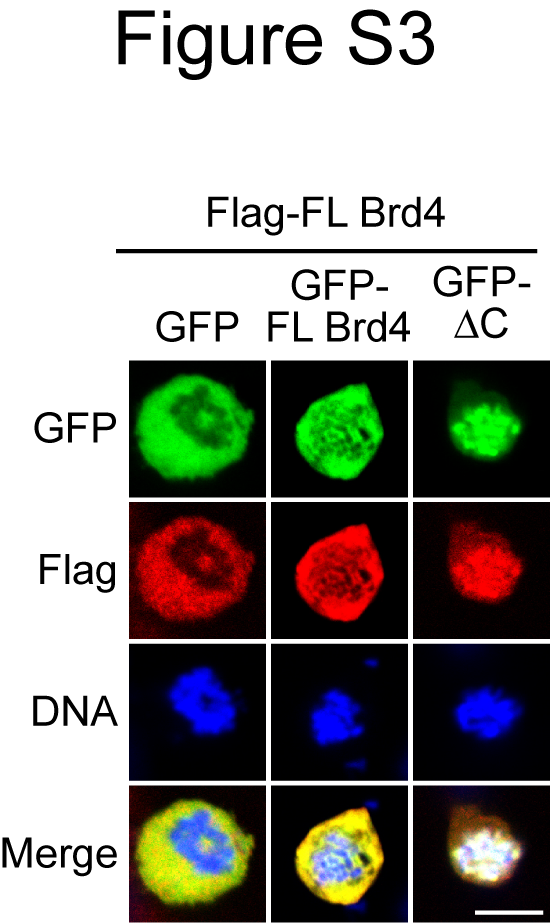

Supplement: Figure S3 — P19 cells were cotransfected withFlag-tagged full length Brd4 along with either GFP alone, GFP-FL Brd4 or GFP-delta C and treated with nocodazole for 4 hours. Cells were fixed immunostained for Flag (red), GFP (green) and counterstained for DNA (blue). (TIF) [file pone.0034719.s003.tif]

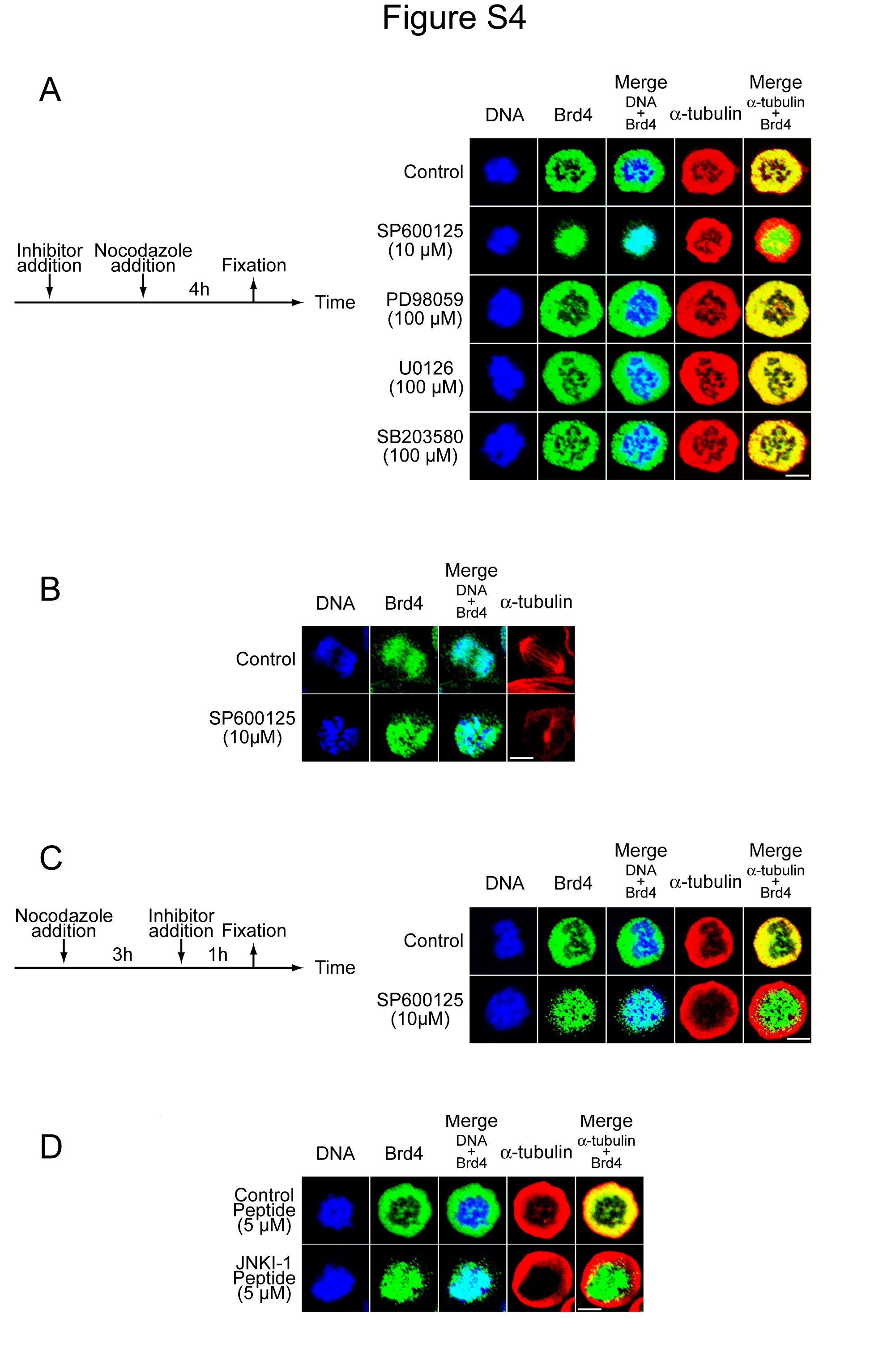

Supplement: Figure S4 — A: JNK inhibitors block nocodazole induced Brd4 release. P19 cells were treated with various inhibitors 30 min or 2 hours prior to the addition of nocodazole. Treatment scheme is shown on the left. Cells were immunostained for localization of Brd4 and tubulin and counterstained for DNA. B: Inhibitors alone do not alter Brd4 localization on Mitotic chromosomes. P19 cells were treated with SP600125 or vehicle control prior to fixation and immunostaining. C: later treatment of inhibitors blocks nocodazole induced Brd4 release. SP600125 was added to cells 3 hours after the start of nocodazole treatment. Treatment scheme is shown on the left. D: JNKI-1 peptide blocks nocodazole induced Brd4 release from mitotic chromosomes. P19 cells were incubated with control or JNKI-1 peptides (5 µM) for 30 min prior to 4 hours nocodazole treatment. After the treatment, cells were immunostained for localization of Brd4, and alpha-tubulin and counterstained for DNA. (TIF) [file pone.0034719.s004.tif]
